# The dose–response characteristics of four NTCP models: using a novel CT-based radiomic method to quantify radiation-induced lung density changes

**DOI:** 10.1038/s41598-020-67499-0

**Published:** 2020-06-29

**Authors:** Dustin Begosh-Mayne, Shruti Siva Kumar, Steven Toffel, Paul Okunieff, Walter O’Dell

**Affiliations:** 1 Lee Memorial Health System, Fort Myers, FL USA; 2 The Department of Radiation Oncology, University of Florida College of Medicine, PO Box 100385, Gainesville, FL 32610 USA

**Keywords:** Computational biophysics, Adverse effects, Computed tomography, Radiotherapy

## Abstract

Multiple competing normal tissue complication probability (NTCP) models have been proposed for predicting symptomatic radiation-induced lung injury in human. In this paper we tested the efficacy of four common NTCP models applied quantitatively to sub-clinical X-ray computed tomography (CT)-density changes in the lung following radiotherapy. Radiotherapy planning datasets and follow-up chest CTs were obtained in eight patients treated for targets within the lung or hilar region. Image pixel-wise radiation dose exposure versus change in observable CT Hounsfield units was recorded for early (2–5 months) and late (6–9 months) time-points. Four NTCP models, Lyman, Logistic, Weibull and Poisson, were fit to the population data. The quality of fits was assessed by five statistical criteria. All four models fit the data significantly (p < 0.05) well at early, late and cumulative time points. The Lyman model fitted best for early effects while the Weibull Model fitted best for late effects. No significant difference was found between the fits of the models and with respect to parameters D_50_ and γ_50_. The D_50_ estimates were more robust than γ_50_ to image registration error. For analyzing population-based sub-clinical CT pixel intensity-based dose response, all four models performed well.

## Introduction

The paradigm of disease treatment is changing. There is an undercurrent moving away from empirical treatment of disease to a personalized approach. The treatment of cancer is at the forefront of this movement. As technology and laboratory techniques improve so does our understanding of biological responses to treatment. A new era of radiomics is emerging as a safe and effective way to measure in vivo treatment effects. The presence of radiomics is felt heavily in the field of radiation oncology. Historically, radiation oncology has relied on treatment protocols for various cancers and target organs. This empirical method will likely shift to biological-based treatment planning. Incorporating radiomics in the investigation and use of treatment models will allow for a more patient specific approach. This approach may lead to dose escalation or de-escalation based on cancer genomics and patient factors such as comorbidities, inflammatory markers, cytokines and other serologies.

Current efforts of radiotherapy and dose escalation are limited by normal tissue complications produced by high-dose radiotherapy^1^. Radiation-induced lung injury such as radiation pneumonitis (RP) and radiation induced lung fibrosis (RILF), collectively known as radiation induced lung injury (RILI), are common complications of radiotherapy of the chest^2,3^. As current therapeutics to treat RP and RILF are lacking, great emphasis has been placed on reducing the probability of developing these lung toxicities. RP is an early effect characterized by inflammation, often appearing during or shortly after radiotherapy and may last upwards of 6 months^4^. Patients often experience shortness of breath, cough, and fever. The risk of developing moderate to severe RP is around 10–20%, however, this depends significantly on the radiotherapy technique and chemotherapy used for treatment^5,6^.

Unlike RP, RILF is a late effect evolving 6–24 months after radiotherapy and is marked by irreversible reduced lung function^7,8^. Symptoms often include a dry cough, chest pain, difficulty breathing, and fatigue. Due to a decreased mucous clearance and obstruction of airways, the fibrosis often worsens and frequently leaves the patient at higher risk for infection^9^. Additionally, RILF is associated with pulmonary hypertension, increasing the risk of developing right heart failure^9^.

The use of dose–response relationships in radiation therapy is crucial in determining the expected outcome for a patient. By establishing the proper normal tissue complication probability (NTCP) or dose response model, treatment plans can be optimized to increase the overall quality of life for patients. Historically the application of NTCP modeling addressed a binary outcome, being presence or absence of a clinical symptom (or survival of a cell, organ or subject) based on pretreatment dose-volume histograms (DVH) and follow-up clinical symptoms^10,11^. These NTCP outcomes typically followed a sigmoid shape and were modeled using two-parameters, with one being D_50_, the dose that results in half of all subjects presenting with a symptom. Several competing models have been developed and although each model represents a sigmoidal curve, each is associated with a different underlying mathematical or biological assumption and the superiority of one over another is a matter of current debate.

Computerized X-ray tomography (CT) scans can provide a more objective and early measurement of RILI^12–15^ over semi-subjective clinical data^16^. Pixel-wise changes in CT Hounsfield unit (HU) can be measured as a function of pixel-wise radiation dose in a graded fashion, where D_50_ is the dose that leads to 50% of maximal HU change for an individual subject. In this paper we compare the efficacy of 4 competing NTCP model formulations, each designed for conventional binary outcomes, in matching pixel-size HU changes. This is a subtle but important distinction from prior art that is necessary to bridge historical knowledge of radiobiological phenomena with state-of-the-art measurement tools.

**Figure 1:**
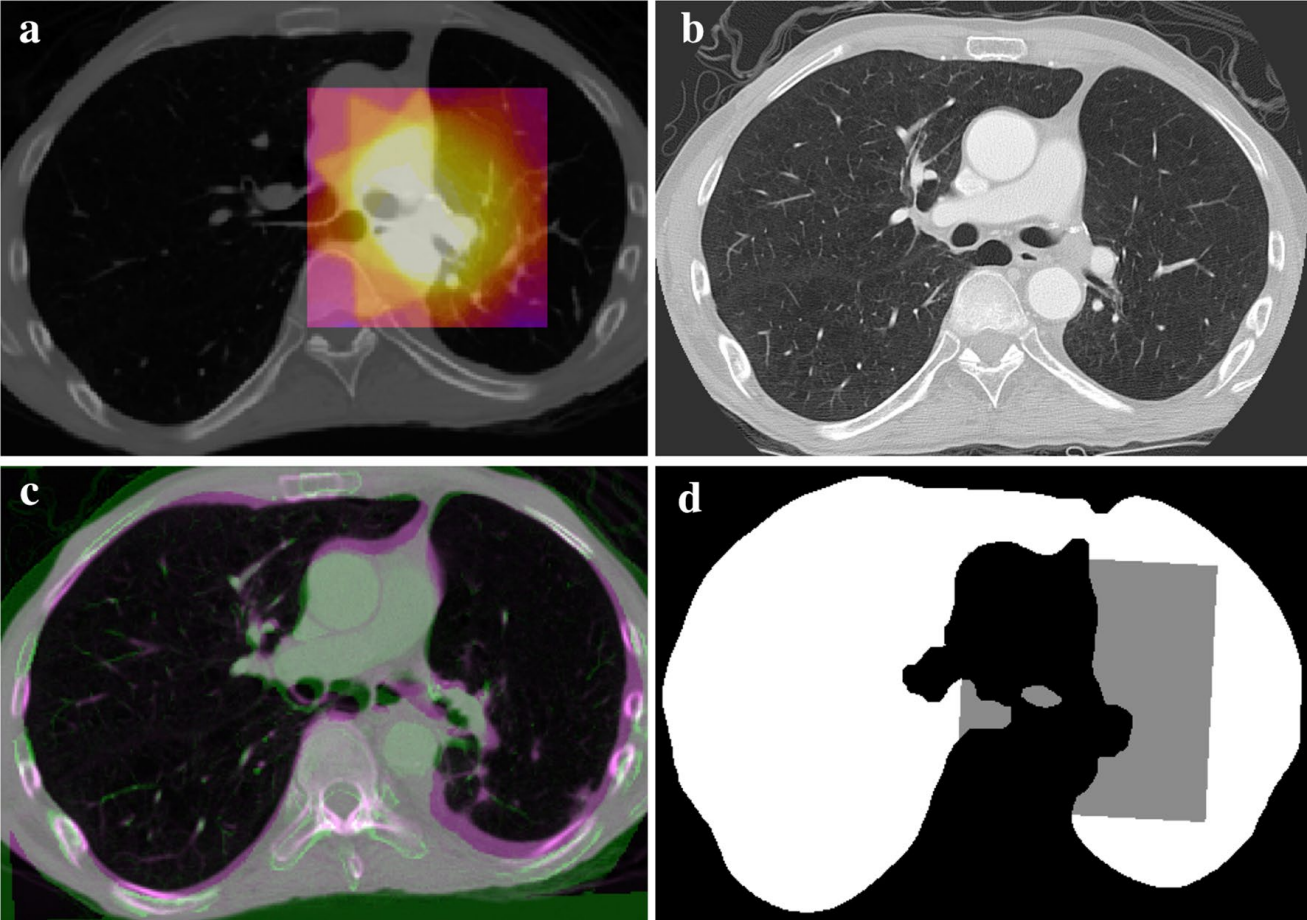
Lung Segmentation and registration results: (**a**) shows for a representative patient a treatment planning chest CT slice through a tumor located within the hilar region. The colorized overlay is the planned dose distribution over a 10 × 10 × 10 cm^3^ region centered on the isocenter. (**b**) An axial CT slice at approximately the same anatomical location at the 8-month follow-up time point and in the same patient as (**a**). (**c**) Depicts the result of the rigid body registration of the 8-month follow-up CT to the panning CT at the same anatomical slice location. (**d**) The lung volume mask in white and gray for the 8-month follow-up image. The computed dose grid is transformed from the planning CT image space to the follow-up CT image space and the gray pixels show where the dose grid aligns to pixels in the lung mask of the follow-up image. The region of gray pixels is where the dose–response data is computed.

Stereotactic body radiation therapy (SBRT) provides a unique opportunity to study the response of normal tissue to radiation. First, SBRT imparts greatly varying doses in the neighborhood of each lesion with almost no radiation to distal sites within the same organ, thereby enabling the determination of full dose/tissue response curves for an individual patient (Fig. 1a). Second, chest CT data sets are often prescribed at multiple time points post-therapy, as per standard of care, providing the ability to monitor the time course of the tissue response. Finally, with the use of lethal radiation doses at the target site there is often no residual tumor mass observed > 3 months post-treatment; when a mass persists, it is generally much reduced in size and ceases to proliferate, suggesting that it remains as a fibrotic core.

## Methods

### Patient and treatment information

Chest CT and radiotherapy planning datasets were obtained (Fig. 1b) retrospectively in patients who were treated with radiation therapy targets within the lung or hilar region at the University of Florida from 2003 to 2013. This study was performed under an UF Institutional Review Board (IRB-)-approved protocol abiding by all institutional, federal and state guidelines. A waiver of informed consent was granted by the IRB for this data collection because this was a retrospective analysis of pre-existing data with minimal risk to subjects. For this study, we separated patients into early CT-changes of 2–5 months and late CT-changes of 6–9 months after completion of radiotherapy treatment. Patients with multiple treatment sites or masses were excluded. Each patient received typically 50 Gy to the clinical target volume in five fractions of 10 Gy/fraction via SBRT. A total of eight patients and 12 follow-up data sets where included in the final analysis: six patients contributing to early changes and six to late changes. All image processing steps were performed using dedicated in-house software written in the Java programming language and built upon the NIH ImageJ platform^17^.

### Dose calculation and CT image acquisition

The radiation dose distributions were computed on a 1 × 1 × 1 mm grid within a ~ 10 × 10 × 10 cm^3^ region, the *dose block*, centered at each planning target volume (Fig. 1a) using the treatment planning system software (Pinnacle^3^ RTP, version 9.2, Philips Medical Systems, Fitchburg, WI, USA). This planning system used the Collapsed Cone Convolution algorithm for dose calculation, which has been deemed clinically acceptable for lung dose calculation^18^. The overall size of the sampled dose field was chosen to encompass the entirety of the 20% isodose line (~ 10 Gy) and to include much of the 10% and lower dose regions. Thus, this dose block included all areas where radiographic and fibrotic changes would be expected to occur, including the D_50_ dose point which is critical for assessing the sigmoidal NTCP models under consideration. The dose values were exported as a DICOM file (‘RTDOSE’ export within Pinnacle^3^ RTP). The CT scans were obtained using a commercial, clinical CT scanner (GE Genesis Lightspeed CT scanner, GE medical system, Milwaukee, Wisconsin) and with imaging parameters consistent with standard-of-care follow-up images at the Department of Radiology at the University of Florida College of Medicine. These parameters were typically an in-plane pixel size of 0.94 mm; slice thickness and slice separation of 3 mm; tube voltage of 120 kVp; and tube current of 250 mA.

### Lung segmentation and image registration

An automatic lung segmentation algorithm was applied to the follow-up CT scans^19^. The algorithm relies on standard histogram-based thresholding and morphological image operations to isolate the lung volume from objects outside the body, the chest wall, diaphragm, and the mediastinum, while including all anatomical features inside the lung border (Fig. 1d). This step omits from consideration pixels outside of the lung volume when computing the dose–response. A three-dimensional (3D) rigid-body registration of the post-radiation treatment (post-RT) to pre-RT data sets was performed (Fig. 1c) using the normalized cross-correlation coefficient as the measure of similarity. The reverse transformation was used to transform the dose field from the planning CT image to each follow-up image. The dose field was sampled on a 1 × 1 × 1 mm grid so that the center of each pixel in the follow-up image was matched to the nearest dose grid point with higher precision. To test the robustness of the outcomes (D_50_, γ_50_) on possible registration mis-match, artificial shifts of plus and minus 3 mm were applied in each superior-inferior, left–right and anterior–posterior direction of the follow-up CT data set with respect to the pre-treatment CT data set in a representative patient data set. The process was repeated for three representative target lesions and the statistics of the dose–response outcomes were tabulated using the standard Lyman model.

### Dose–response calculations

Radiation dose exposure versus change in observable CT Hounsfield units (HU) was calculated for various time-points post-radiation therapy. Change in CT image intensity was computed for each lung pixel within the dose-block in the follow-up CT image set. Dose values were binned into 6 Gy-wide bins from 0 to 60 Gy, and the average CT intensity change was computed over all pixels within each bin. Change in CT image intensity was computed by subtracting the intensity in each pixel from the baseline intensity value within the dose-block. The change was then normalized to a range of 0–1 by scaling to the greatest change in any dose bin (typically the 54–60 Gy bin), reflective of the maximal possible response for that patient. Dose–response data points for each individual patient data set were generated in this manner and fit to each NTCP model. The models were fit using the generalized reduced gradient method within Excel (Microsoft Excel for Mac, Version 16.36) which employs an iterative least square fitting routine to produce the optimal goodness of fit between data and function^20^. The follow-up CT scans from eight patients were segregated into six scans obtained between 2 and 5 months post-radiation therapy (post-RT), typically representative of the time of presentation of RP, and six scans obtained between 6- and 9-months post-RT, typically representative of the time of presentation of RILF.

To compare the D_50_ and γ_50_ values across the NTCP models, fits were first made individually to each of the six patient data sets at the early and late time-points to compute the average and range of D_50_ and γ_50_ values for each time-range. The data sets for all 12 times were then combined and fit to compute the cumulative average and range of D_50_ and γ_50_ values (Table 1).

**Table 1:** D_50_ and γ_50_ values.

Name of model	D_50_ (Gy)	γ_50_	SSR	Adj-R^2^	AIC	BIC
2–5 months						
Logit	33.72 (25.97–47.96)	1.76 (0.65–3.93)	0.14	0.92	− 22.65	− 22.04
Lyman^a^	34.73 (27.40–47.73)	1.38 (0.66–3.36)	0.07	0.95	− 29.16	− 28.56
Weibull	34.49 (26.68–47.98)	1.40 (0.65–3.48)	0.08	0.95	− 28.26	− 27.66
Poisson	33.78 (26.10–47.48)	1.42 (0.62–3.66)	0.09	0.94	− 26.62	− 26.02
6–9 months						
Logit	35.13 (22.46–44.07)	0.77 (0.23–1.13)	0.15	0.88	− 21.83	− 21.23
Lyman	35.85 (23.82–43.64)	1.32 (0.65–2.94)	0.13	0.90	− 23.58	− 22.98
Weibull^a^	35.69 (23.3–43.67)	1.32 (0.54–2.79)	0.13	0.90	− 23.63	− 27.66
Poisson	35.20 (22.79–43.91)	1.78 (0.60–5.93)	0.22	0.85	− 18.16	− 17.56
Cumulative						
Logit	34.43 (22.46–47.96)	1.26 (0.23–3.93)	0.11	0.91	− 25.02	− 24.41
Lyman^a^	35.29 (23.82–47.73)	1.35 (0.65–3.36)	0.09	0.93	− 26.54	− 25.93
Weibull	35.09 (23.3–47.98)	1.36 (0.54–3.48)	0.10	0.93	− 26.24	− 25.64
Poisson	34.49 (22.79–47.48)	1.60 (0.60–5.93)	0.15	0.90	− 21.94	− 21.34

Population average and range values (in parenthesis) for D_50_ and γ_50_ at any given time point were calculated for Logit, Lyman, Weibull, and Poisson models to the six data sets at each time frame. D_50_ is the dose at which 50% of maximal CT HU change was expected to arise, and γ_50_ is the normalized dose response gradient at the D_50_ level. Also shown here are the computed sum of squared residuals (SSR), Adjusted R^2^ (Adj-R^2^), Akaike information criterion (AIC) and the Bayesian information criterion (BIC) for each model fit.

^a^The best fit model based on the lowest SSR, AIC and BIC and the highest adj-R^2^.

**Figure 2:**
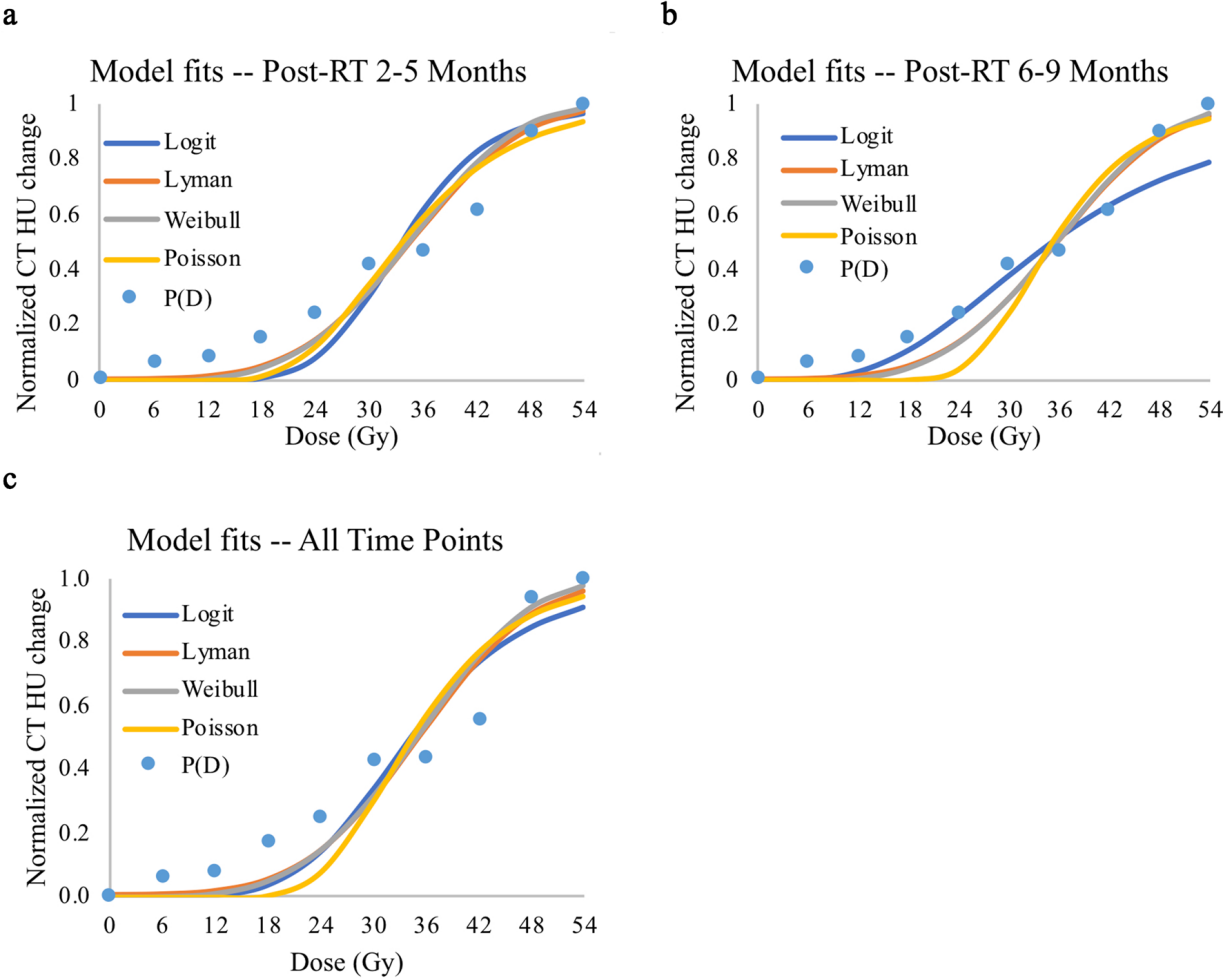
NTCP modeling of radiation-induced effects to the lung: dose response data and model-fits for stereotactic body radiation therapy-induced injury of the lungs. Blue dots (P(D)) represent the radiation-induced effects, defined as the cumulative change in CT HU over all associated patients, normalized from 0 to 1 based on the maximal CT HU change seen at the particular time point. Blue, orange, gray, and yellow lines represent the Logit, Lyman, Weibull, and Poisson dose response models, respectively, fit to the data points. (**a**) The data and fits of six patients at 2–5 months after radiation therapy. These time points reflect radiation pneumonitis. (**b**) The data and fits of six patients at 6–9 months after radiation therapy. These time points reflect radiation-induced lung fibrosis. (**c**) The data and fits of all 12 patient data sets over all time points.

Next, to compare the quality of the model fits, for each dose bin the patient-normalized change in CT intensity values was averaged across all six patients for each time-point to generate a cumulative dose–response data set. The cumulative data was normalized to the largest intensity in any bin in any of the patients and the NTCP models were then fit to these cumulative dose–response data (Fig. 2).

### Dose response models for normal tissue complication probability

For this paper, we compare the four of the most commonly-implemented models traditionally used for modeling incidence-based lung tissue response to radiation (i.e. the presence or absence of clinical RP versus mean lung dose): Lyman, Logit, Weibull and Poisson^11,22^. Each is based on a different statistical distribution, being the Probit (Gaussian), Logistical, Weibull and Poisson, respectively. The Poisson model is cell-survival-based, while the rest are phenomenological^21^. Each model may be expressed in terms of the parameters D_50_ and γ_50_. Here, D_50_ is the dose necessary to cause 50% maximal HU change, and γ_50_ is the gradient of the dose–response curve at the level of the 50% toxicity. A detailed derivation of each model is outside the scope of this paper but may be found in works done by the Karolinska Institute group and others^21,23^.

The Lyman model is based on the cumulative distribution function of the dose-dependent Gaussian distribution^24^. The integral form representation is:

1 $$P\left(D\right)= \frac{{\gamma }_{50}}{{D}_{50}}{\int }_{0}^{D}{e}^{{-\frac{1}{\pi }\left[\gamma \frac{x-{D}_{50}}{{D}_{50}}\right]}^{2}}dx$$

This function can be solved using a Probit function represented as:

2 $$P\left(D\right)=0.5\left(1-erf\left[{\gamma }_{50}\sqrt{\pi }\left(1-\frac{D}{{D}_{50}}\right)\right]\right)$$

The parallel architecture model, or logit model, is commonly applied to biological processes and is based on the cumulative distribution function of the logistic function:

3 $$P\left( D \right) = \frac{1}{{1 + \left( {\frac{{D_{50} }}{D}} \right)^{k} }};\quad \gamma_{50} = \frac{1}{4}k$$

The Poisson model developed by the Karolinska Institute group^21,23^ has a radiobiology background and is based on Poisson statistical model of cell death and is represented as:

4 $$P\left(D\right)={2}^{-\mathrm{e}\mathrm{x}\mathrm{p}\left[e\gamma \left(1- \frac{D}{{D}_{50}}\right)\right]}$$

The Weibull model is based on a modified Weibull function. Like the Poisson model, the Weibull model is biologically based. The cumulative distribution function for the Weibull distribution has been used as a mathematical scaffold for several theories of cell death and survival^25–27^. The Weibull model is represented as:

5 $$P\left(D\right)=1-exp\left[-\mathrm{l}\mathrm{n}2{\left(\frac{D}{{D}_{50}}\right)}^{\frac{2}{\mathrm{l}\mathrm{n}2}{\gamma }_{50}}\right]$$

To calculate the parameters D_50_ and γ_50_ the experimental data was normalized to the HU units of the maximum dose bin to fit a probabilistic model (bin dose value versus bin-averaged CT intensity changes). Once normalized each model was solved using generalized reduced gradient method.

### Statistical methods

To assess the quality of fit, a one-way Analysis of Variance (ANOVA) was used to test for significance of each model fit to the data. The quality of fits was compared by the computing sum of squared residuals (SSR), Adjusted R^2^ (adj-R^2^), Akaike information criterion (AIC)^28^ and the Bayesian information criterion (BIC)^29^. While SSR is commonly used, it is biased toward models with more parameters. Adj-R^2^, AIC and BIC incur penalties for high parameter number. BIC is best for identifying the true model among a pool of candidate models. AIC is best for identifying the best available model among a pool of imperfect models^30^. Here, we compared the models based on low SSR, AIC and BIC, and high adj-R^2^ values. We did this for the cumulative data for all six early time-point data sets, all six late-time frame data sets, and for the cumulative (all 12) data sets (Table 1). An analysis of variance (ANOVA) was used to investigate any statistically significant differences between the fitted models and the parameters D_50_ and γ_50_ between the models.

## Results

### Model comparison

An ANOVA revealed that all models individually fitted the true data significantly (p < 0.05) well for early effect, late effect and for the cumulative time points. In the model fit comparison, Lyman model outperformed other models for both early effects (SSR: 0.07, AIC: − 29.16, BIC: − 28.56, adj-R^2^: 0.95) and over all time-points (SSR: 0.09, AIC: − 26.54, BIC: − 25.93, adj-R^2^: 0.93) (Table 1), however, the Weibull model was the best for late effects (SSR: 0.13, AIC: − 23.63, BIC: − 23.03, adj-R^2^: 0.90) (Table 1). An ANOVA did not find any significant differences (p > 0.05) between any of the four models when determining NTCP for early effect, late effect and for the cumulative time points.

### ***Comparison of D***_***50***_*** and ***${{\varvec{\gamma}}}_{50}$

The D_50_ and γ_50_ of all models were examined (Table 1 and Fig. 2). One-way ANOVA found no significant difference between the models with respect to D_50_ and γ_50_. The Weibull model exhibited the highest D_50_ for CT scans obtained at 2–5 months follow-up while the Logit model exhibited the highest D_50_ for CT scans at 6–9 months follow-up (no significant difference). The Logit dose response model produced the lowest D_50_ for all data sets (no significant difference) and exhibited the highest γ_50_ for CT scans at 2–5 months. The Poisson model exhibited the highest γ_50_ for CT scans at 6–9 months (no significant difference). The Logit dose response model produced the lowest γ_50_ for all datasets (no significant difference).

### Robustness to error in image registration

Table 2 summarizes the changes in D_50_ and γ_50_ using the Lyman model (Eq. 2) after applying, plus and minus 3 mm shifts individually in each superior-inferior, left–right and anterior–posterior direction. The process was repeated for three different target lesions and their dose–response outcomes, labeled Targets 1–3 in the table. For each target, the maximum error over any of the six trials (two shifts in each of three directions) was computed and converted to a percentage change from the baseline values computed from the optimal registration. The D_50_ value had a maximal change over all three targets of only 4.4% for a 3-mm error in registration. The γ_50_ parameter was much more sensitive to registration error, with a maximal change over all three targets of 180% for a 3-mm registration shift.

**Table 2:** Effect of image registration errors on D_50_ and γ_50_ values.

Target	1	2	3
Reference D_50_	52.12	51.67	42.10
D_50_ error max	0.9%	4.4%	2.7%
Reference γ_50_	12.31	6.99	10.7
γ_50_ error max	10%	180%	12%

Effect of imposing a ± 3 mm registration error in each superior-inferior, left–right and anterior–posterior direction on the dose response parameters: D_50_ and γ_50_. The D_50_ is robust to registration errors up to 3 mm in any direction, while γ_50_ is very sensitive to such error.

## Discussion

Patient-specific dose–response curves for changes in tissue density have been generated from serial CT scans of patients receiving locally high radiation dose for treatment of cancer to or near the lung. The approach involves registering a follow-up CT scan to the CT scan used for creating the radiation treatment plan; exporting the planned 3D dose field and transforming it to the follow-up time point using the registration information; segmenting the lung volume in the CT images; computing, for each pixel in the lung, the dose received during treatment and the normalized change in CT pixel intensity at the follow-up time; segregating the pixels by dose into 6-Gy bins; and plotting the pixel-averaged change in CT intensity as a function of dose-bin.

The primary objective of this paper was to evaluate which of the four most-common NTCP models best represent the CT-based dose–response data of pulmonary tissue for a population of eight patients and establish the superiority of one model over the other in predicting. The most commonly used NTCP model for predicting RILI is the Lyman model which is purely mathematical and fits the data using an underlying Gaussian distribution. The aim of this paper was to evaluate whether the cell-survival based models (Poisson) or other mathematically based ones (Logit and Weibull) perform almost as well or even better that the Lyman model in predicting NTCP. Our analysis showed that all the models statistically fit the true data well (p < 0.05) and there were no statistically significant differences in the NTCP predictive capability between any of the four models (p > 0.05).

However, on comparing the individual fits using parameters such as the SSR, adj-R^2^, AIC and BIC, the Lyman model performed best for fitting early changes (3–6 months) and for cumulative time points while the Weibull model performed best for fitting late changes (6–9 months). A second objective was to establish the consistency in the fitted values for the two model parameters, D_50_ and γ_50_, that are common for all four models. When using the Lyman model, D_50_ was robust to registration errors of up to 3 mm while γ_50_ was very sensitive to registration error. Across all four models, no statistically significant differences in the parameter values was found with our relatively small sample set. Future studies with additional subjects may be needed to discern whether a small but significant difference in parameter values and model fits exist.

The estimated D_50_ and γ_50_ values in this study (range 33.7–40.4 for D_50_, and 0.77–1.78 for γ_50_) conforms to previously computed values in the literature. The QUANTEC (quantitative analysis of normal tissue effects in the clinic) data^31^ estimated D_50_ as 30.8 Gy (95% CI 28.7, 33.9) and γ_50_ as 0.97 (95% CI 0.83, 1.12). Appelt et al.^31^ computed the baseline D_50_ and γ_50_ in an independent dataset consisting of 103 patients and found the reference D_50_ and γ_50_ for a patient without pulmonary co-morbidities, caudally located tumor, no history of smoking, < 63 years old, and receiving no sequential 34.4 Gy (95% CI 30.7, 38.9) and 1.19 (95% CI 1.00, 1.43) respectively. Wedenberg et al.^32^ estimated D_50_ as 32.4 Gy (95% CI 28.9, 37.3) and γ_50_ as 0.57 (95% CI 0.47, 0.68) using a Poisson-based model, and D_50_ as 31.4 Gy (95% CI 28.5, 35.6) and γ_50_ as 0.49 (95% CI 0.42, 0.58) using a Lyman based model. However, the parameters values found in this SBRT study relate to tissue density changes observed within individual patients in the presence of steep dose gradients^33,34^, while those of the prior art relate to incidence of clinical symptoms across patients receiving conventional, large-field lung RT. The dose-volume characteristics of SBRT being quite different from conventional lung RT, as well as there being differences in the biological mechanisms and the treatment planning systems over time, caution should be used when attempting to extend the parameters values from in this study to patients receiving different treatments and for different outcomes.

**Figure 3:**
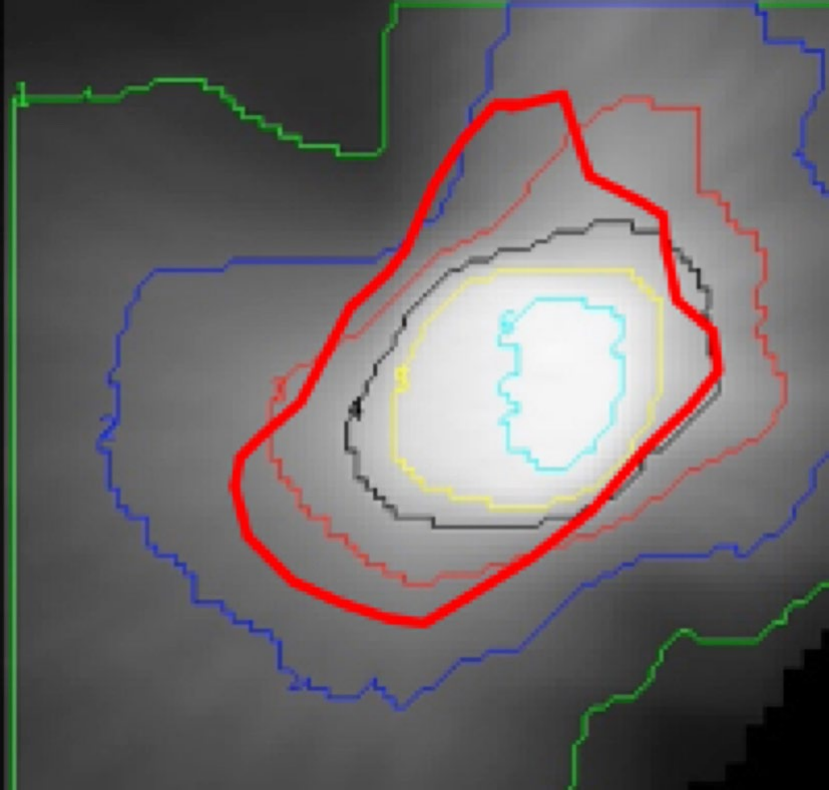
Robustness to error in image registration: the effect of mis-registration on the dose–response D_50_ and γ_50_ outcomes. Shown is the 3-dimensional dose field for an SBRT target, with isodose contours overlaid onto the gray-scale dose image. Registration error results in a shift of the d_50_ contour (bold red curve). Since the new D_50_ estimate is the average dose along the bold red curve and the new curve crosses both higher and lower dose regions, the d_50_ value may not change dramatically. However, since γ_50_ represents the heterogeneity in response along the D_50_ contour, its value is very sensitive to registration error.

The explanation for the robustness of D_50_ to induced registration error is diagrammed in Fig. 3. If the thin maroon curve in Fig. 3 represents the D_50_ isodose contour (in an ideal scenario), then registration error results in a shift of this D_50_ contour with respect to the true dose-distribution around the target lesion (to the bold red curve). The new estimate for D_50_ is directly related to the average dose along the new red curve. For the case where the tissue response is a monotonic function of dose, the average lung tissue response along this curve also may not change appreciably. However, the parameter γ_50_ reflects the heterogeneity in response at the D_50_ dose contour and since the shifted D_50_ contour now traces over pixels experiencing a wider range of doses, the tissue response along the new D_50_ contour is much more heterogeneous. For this reason, it is recommended to use D_50_ as the indicator of choice when measuring tissue response when there is concern for inaccurate registration.

Diot, et al.^15^ reported the change in pixel-wise CT HU versus regional dose in 62 patients receiving SBRT at 3, 6, 12, 18, 24, and 30 months post-treatment. They observed an increase in CT density over time to around 6 months, followed with a less pronounced late response. They also reported an increase in the CT HU with dose, up to 35 Gy, and a plateau at higher doses. Underwood et al.^26^ and Li et al. ^31^ compared CT density changes over time between proton therapy and traditional X-ray-based therapy, for breast cancer and NSCLC patients, respectively. Each of these three prior studies, however, used a linear model (fitted regression line) to what is expected to be an intrinsically s-shaped dose–response curve. The current paper extends these prior efforts by comparing the accuracy of the four most-common and historically supported sigmoid-shaped NTCP models. Moreover, the current paper establishes model parameter robustness as a first step in the greater task of elucidating the biological and mechanistic underpinnings of the dose-HU response.

The biological link between radiographic changes and radiation response is not fully understood. It is likely though that early changes in HU during the radiation pneumonitis phase are dominated by a local inflammatory response and damage to microvascular endothelial cells leading to vessel leakiness^35^. HU changes seen at later time points likely reflect radiation-induced fibrosis^2–5^ and lung tissue consolidation^36^.

The association between the clinical manifestation of RILI and the measurement of radiographic changes is also a topic of great interest. There are several challenges in making this connection. First, the underlying biological phenomenon are not necessarily the same and not currently fully understood. Second, additional treatment and patient factors may impact them differently. These include the volume of irradiated tissue, the fractionation scheme, a patient’s smoking history and presence of comorbidities. Also, as noted above, clinical RILI is primarily a binary outcome with subjective interpretation, whereas change in CT HU is a continuous variable that can be measured objectively. The challenges are hoped to be addressed in the future in larger and more longitudinal studies.

It is hoped that additional studies focusing on quantifying subclinical markers of radiation injury will enable us to identify high-risk patients early during treatment to permit treatment modification or early intervention to mitigate or delay the progression of RILI.

## Conclusion

Using population-averaging of patient-specific full dose–response curves for pulmonary tissue from serial chest CT scans, we have determined that all the four models: Lyman, Logit, Weibull and Poisson, fit the data well (p < 0.05) however, no statistically significant differences existed between the fits of the four models for early, late and cumulative time-point effects. On assessing the fits to individual subjects, the Lyman NTCP model (SSR: 0.07, AIC: − 29.16, BIC: − 28.56, adj-R^2^: 0.95) fit better than the Logit, Weibull and Poisson NTCP models when considering only early effects (2–5 months post-treatment) while the Weibull model (SSR: 0.13, AIC: − 23.63, BIC: − 23.03, adj-R^2^: 0.90) fit best for late effects (6–9 months post-RT). Additionally, the resulting D_50_ and γ_50_ parameter values were not significantly different between any of the 4 NTCP models tested. Therefore, while these models have different cell-survival or mathematical motivations, for the analysis of CT pixel intensity-based dose response, either of the four models tested could be used to perform this assessment. The estimated D_50_ values were found to be robust to image registration errors up to 3 mm (the largest shifts that were tested) while the γ_50_ values were highly sensitive to registration error. Thus, for modeling of radiation sensitivity it is recommended that the D_50_ value be used rather than relying solely on γ_50_. Additional work is needed to ascertain which NTCP model is best to distinguish differences in radiation sensitivity across patients, over time, or in response to intervention.
